# Influence of Vitamin D on the Vasoactive Effect of Estradiol in a Rat Model of Polycystic Ovary Syndrome

**DOI:** 10.3390/ijms22179404

**Published:** 2021-08-30

**Authors:** Róbert Tarszabó, Bálint Bányai, Éva Ruisanchez, Borbála Péterffy, Ágnes Korsós-Novák, Krisztina Lajtai, Réka Eszter Sziva, Dóra Gerszi, Ádám Hosszú, Rita Benkő, Zoltán Benyó, Eszter Mária Horváth, Gabriella Masszi, Szabolcs Várbíró

**Affiliations:** 1 Department of Obstetrics and Gynecology, Markusovszky Lajos University Teaching Hospital, Markusovszky Lajos Street 5, 9700 Szombathely, Hungary; 2 Department of Physiology, Semmelweis University, Tűzoltó Street 37-47, 1094 Budapest, Hungary; banyai.balint@gmail.com (B.B.); peterffy.borbala@gmail.com (B.P.); krisztina.lajtai@gmail.com (K.L.); sziva.reka@semmelweis-univ.hu (R.E.S.); dora.gerszi@gmail.com (D.G.); benko.rita@med.semmelweis-univ.hu (R.B.); horvath.eszter@med.semmelweis-univ.hu (E.M.H.); 3 Department of Translational Medicine, Semmelweis University, Tűzoltó Street 37-47, 1094 Budapest, Hungary; ruisanchez.eva@med.semmelweis-univ.hu (É.R.); benyo.zoltan@med.semmelweis-univ.hu (Z.B.); 4 Department of Pathology, Hetényi Géza Hospital, Tószegi Street 21, 5000 Szolnok, Hungary; novak.agnes@gmail.com; 5 Department of Obstetrics and Gynecology, Semmelweis University, Üllői Street 78/a, 1082 Budapest, Hungary; varbiro.szabolcs@med.semmelweis-univ.hu; 6 1st Department of Pediatrics, Semmelweis University, 1082 Budapest, Hungary; hosszu.adam@gmail.com; 7 Department of Internal Medicine, National Institute of Psychiatry and Addictions, Lehel Street 59-61, 1135 Budapest, Hungary; gabriellamasszi@gmail.com

**Keywords:** polycystic ovary syndrome, vitamin D deficiency, rat model, estradiol relaxation, testosterone treatment

## Abstract

We examined the vasoactive effect of estradiol in a rat model of early PCOS and the influence of vitamin D deficiency (VDD). We created a model of chronic hyperandrogenism and VDD in adolescent female Wistar rats (N = 46) with four experimental groups: vitamin D supplemented (T-D+), VDD (T-D-), hyperandrogenic and vitamin D supplemented (T+D+), and hyperandrogenic and VDD (T+D-). T+ groups received an 8-week-long transdermal Androgel treatment, D-animals were on vitamin D-reduced diet and D+ rats were supplemented orally with vitamin D3. Estrogen-induced vasorelaxation of thoracic aorta segments were measured with a wire myograph system with or without the inhibition of endothelial nitric oxide synthase (eNOS) or cyclooxygenase-2 (COX-2). The distribution of estrogen receptor (ER), eNOS and COX-2 in the aortic wall was assessed by immunohistochemistry. VDD aortas showed significantly lower estradiol-induced relaxation independently of androgenic status that was further decreased by COX-2 inhibition. COX-2 inhibition failed to alter vessel function in D+ rats. Inhibition of eNOS abolished the estradiol-induced relaxation in all groups. Changes in vascular function in VDD were accompanied by significantly decreased ER and eNOS staining. Short-term chronic hyperandrogenism failed to, but VDD induced vascular dysfunction, compromised estrogen-dependent vasodilatation and changes in ER and eNOS immunostaining.

## 1. Introduction

Polycystic ovary syndrome (PCOS) affects approximately 2–20% of women in reproductive age which makes it the most prevalent endocrine disease of this cohort [1]. PCOS can be genetically determined but it depends greatly on environmental influences. Diagnosis of PCOS relies on the Rotterdam criteria: two of the following three should be observed: clinical or biochemical hyperandrogenism, ovulatory dysfunction and polycystic morphology of the ovaries [2]. The early complications are menstrual irregularity, infertility, hirsutism and acne. Later co-morbidities present as part of metabolic syndrome including type 2 diabetes, hypertension and other cardiovascular diseases (CVD) [3,4].

The early appearance of vascular dysfunction in the pathogenesis of the disease may contribute to the increased CV risk [3]. Deterioration of endothelium-dependent vasorelaxation can be the first sign of vascular dysfunction [5]. The lower prevalence of hypertension in women of reproductive age might be related to the vasorelaxant effect of estrogen [6], and the higher risk of hypertension in women with PCOS could be caused by the impairment of this mechanism [7].

It is well known that there are several non-skeletal chronic diseases, which are linked to vitamin D deficiency e.g., metabolic disorders, cardiovascular diseases, infectious diseases and cancer [8]. Lower levels of serum vitamin D are commonly associated with PCOS which suggests a possible role in the pathomechanism of the disease [9,10]. Vitamin D deficiency itself can result in endothelial dysfunction [11]. A key player in adequate endothelial function is eNOS function. Vitamin D can activate eNOS not only through the increase of intracellular calcium levels but via the PI3K/Akt pathway [12]. Prior studies reported that vitamin D deficiency decreased eNOS level and vasorelaxation in rat renal arteries but vitamin D treatment was able to improve acetylcholine-induced relaxation [13].

Prostaglandins (PG) are produced from arachidonic acid by cyclooxygenase (COX) enzymes. It was shown that the amount of mainly COX-2 derived metabolites PGJ_2_, PGE_2_, PGF_2α_, and thromboxane B_2_ [TXB_2_]) is increased in the follicular fluid of PCOS women. The concentration of these compounds had a significant correlation with serum basal testosterone levels. The increased level of these prostanoids in the inflammatory environment may play role in the emerging ovarian dysfunction.

In our previous study, we demonstrated reduced estrogen-induced relaxation and increased oxidative stress in isolated aortic rings of dihydro-testosterone (DHT) treated rats. The chronic treatment in that setting lasted 10 weeks. Vitamin D administration partially ameliorated these changes [14]. In case of a shorter 8-week-long testosterone treatment, a decreased acetylcholine-mediated vasorelaxation was detected in the vitamin D deficient group, and the same effect was observed as the insulin–mediated vasorelaxation was measured [15].

The aim of the present study was to examine the vasodilatory effect of estradiol and to elucidate the underlying mechanism, at an even earlier phase of hyperandrogenic state and better observe the possible influence of vitamin D deficiency.

## 2. Results

### 2.1. PCOS Characteristics in Our Model

PCOS-like characteristics were confirmed in our model and published by Hadjadj et al. in 2018 [16]. Briefly, following testosterone treatment, a 10-fold increase in serum testosterone, and a 5-fold increase in DHT levels were registered, while androgen levels of non-treated animals were in the normal range. Vitamin D deficiency (serum levels under 20 ng/mL) was achieved by vitamin D-reduced diet, meanwhile, vitamin D supplementation was able to provide target serum vitamin levels of 30–50 ng/mL [17,18]. Histological examinations showed polycystic morphology of the ovaries in the testosterone-treated groups, having an increased number of primordial follicles due to missing ovulation. Cervical smear analysis confirmed that these animals had no estrus cycle [16].

### 2.2. Estradiol Induced Relaxation of Isolated Thoracic Aorta Segments

**Figure 1:**
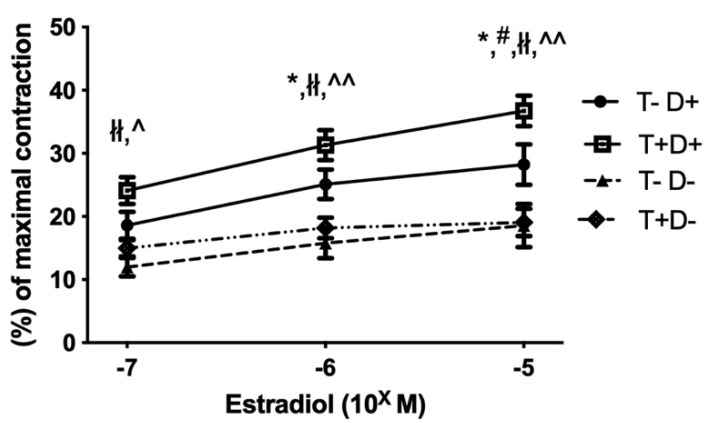
Estradiol dependent vasorelaxation. All precontracted thoracic aorta segments dilated as a result of increasing concentrations of estradiol. In the vitamin D deficient groups, significantly reduced estradiol-induced vasorelaxation was measured, which seemed to be independent of the androgen status. Data are presented as mean ± SEM. *: *p* < 0.05 T-D+ vs. T-D-, #: *p* < 0.05 T-D+ vs. T+D-, łł: *p* < 0.01 T+D+ vs. T-D-, ^: *p* < 0.05 T+D+ vs. T+D-, ^^: *p* < 0.01 T+D+ vs. T+D-.

**Figure 2:**
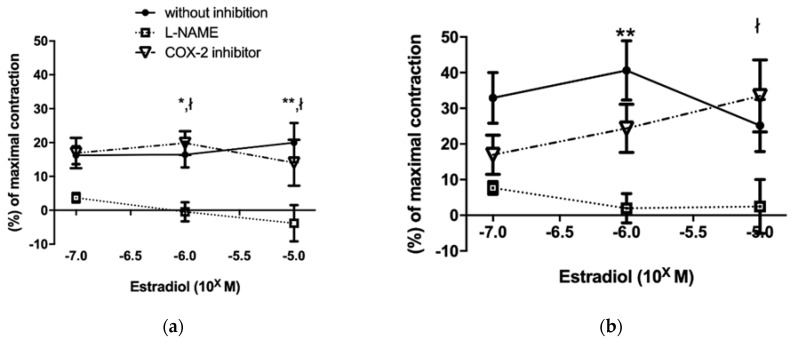

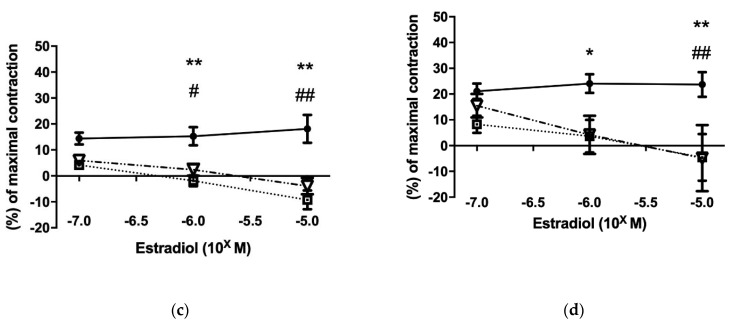
Estradiol-induced vasorelaxation in the presence of eNOS (L-NAME) or COX-2 (NS398) inhibitors. Panel (**a**): T-D+ group. Panel (**b**): T+D+. Panel (**c**): T-D-. Panel (**d**): T+D-. After L-NAME incubation, estradiol dependent vasorelaxation significantly decreased in each group. After incubation with COX-2 inhibitor, vasorelaxation decreased only in the vitamin D deficient groups but testosterone treatment did not influence it. Data are presented as mean ± SEM. Repeated measures two-way ANOVA using Bonferroni’s post hoc test: *: *p* < 0.05 L-NAME vs. without inhibition, **: *p* < 0.01 L-NAME vs. without inhibition, ł: *p* < 0.05 L-NAME vs. COX-2 inhibitor, #: *p* < 0.05 COX-2 inhibitor vs. without inhibition, ##: *p* < 0.01 COX-2 inhibitor vs. without inhibition.

The increasing concentration of estradiol (10^−7^–10^−5^ M) caused the relaxation of precontracted (5 × 10^−8^ norepinephrine) thoracic aorta segments in all experimental groups. Aortic segments of VDD rats showed significantly lower estradiol-induced relaxation independently of androgenic status (Figure 1). The reduced estradiol-dependent relaxation of vitamin D deficient aortas was further decreased by COX-2 inhibition, whereas COX-2 inhibition had no effect on the vessels of vitamin D supplemented rats (Figure 2 Panel (a,b)). Inhibition of eNOS by pretreatment with L-N^G^-Nitro arginine methyl ester (L-NAME) abolished the estradiol-induced relaxation in all experimental groups. (Figure 2 Panel (a–d)).

### 2.3. Immunohistochemistry

**Figure 3:**
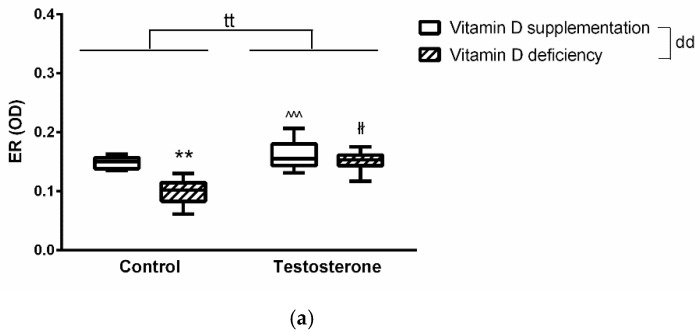

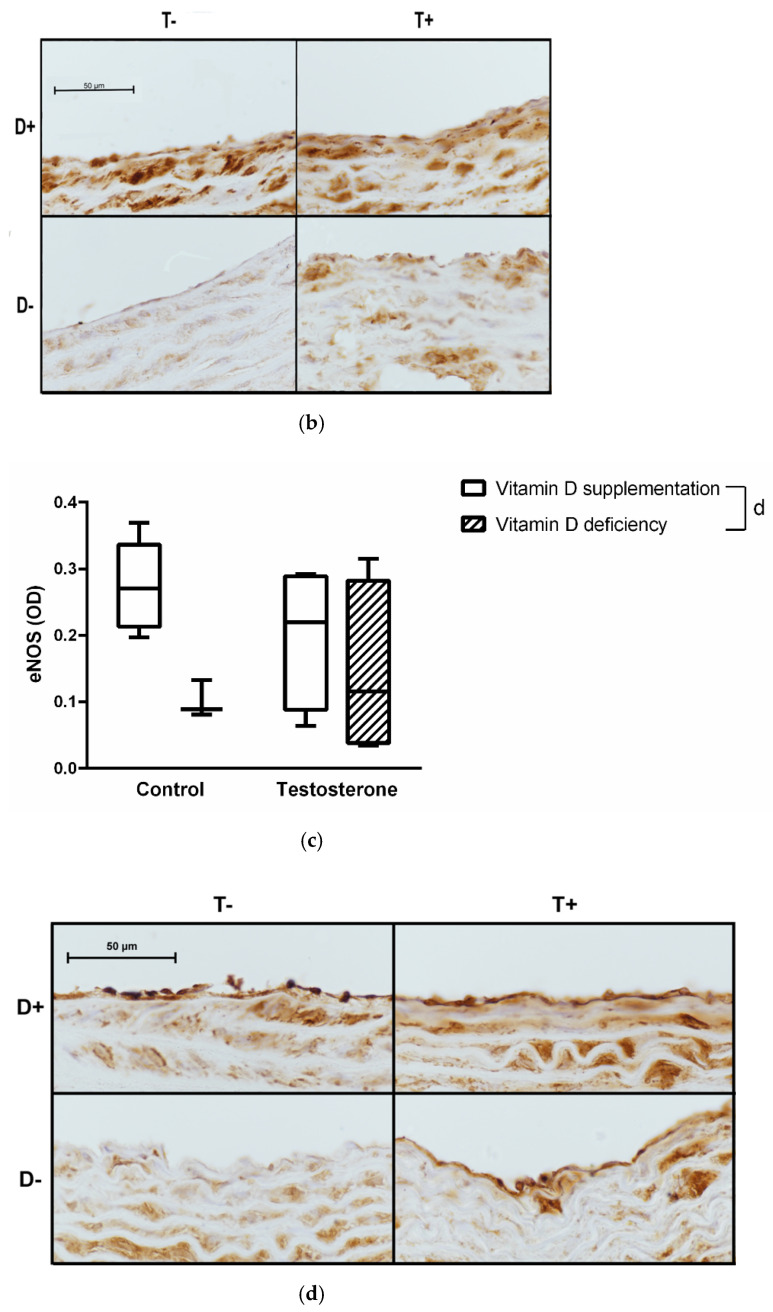

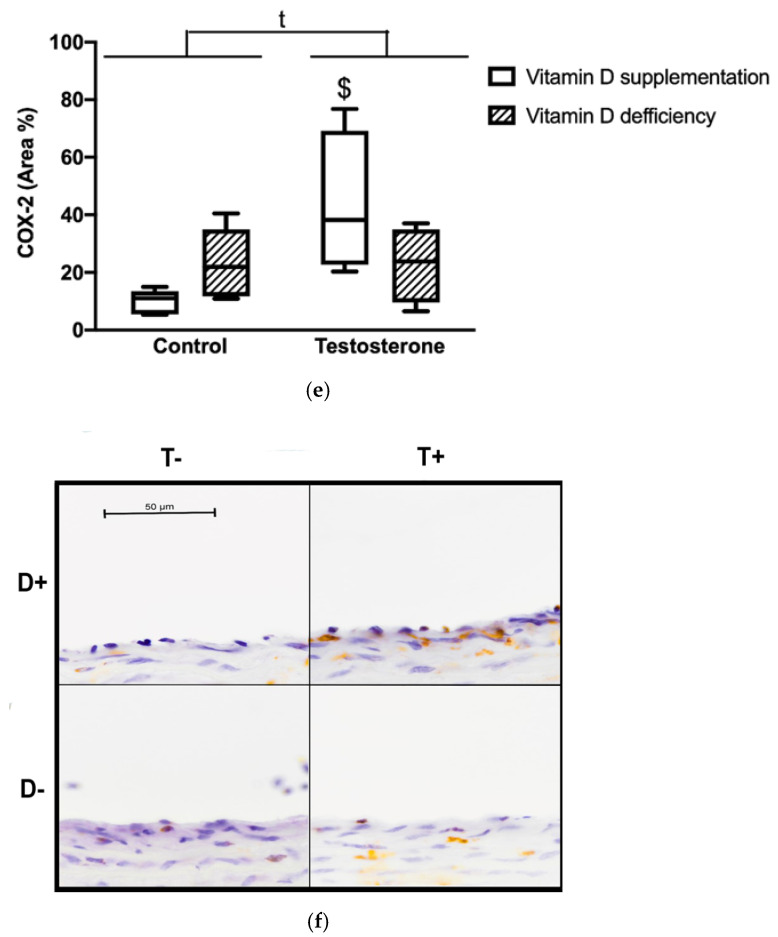
Immunohistochemical changes in thoracic aorta wall. Panel (**a**)**.** Immunohistochemical staining of the aortic wall with ERα antibody. Non-calibrated optical density was measured. Vitamin D deficiency significantly decreased while testosterone treatment increased ERα specific staining. T-D- group had significantly lighter staining compared to all other experimental groups. Panel (**b**) shows representative images of ERα staining. Specific staining is visualized by brown-colored DAB, while blue-colored hematoxylin serves as counterstaining. Data are presented as mean ± SEM. Two-way (testosterone treatment and vitamin D status) ANOVA; Tukey’s post-hoc test, **: *p* < 0.01 T-D+ vs. T-D+ group, ^^^: *p* < 0.005 T-D+ vs. T+D+ group, łł: *p* < 0.01 T-D- vs. T+D- group; tt: *p* < 0.01 T- vs. T+, dd: *p* < 0.01 D+ vs. D-. N = 5–6 in each group. Panel (**c**). Immunohistochemical staining of the aortic wall with eNOS antibody. Vitamin D deficiency significantly decreased eNOS specific staining while testosterone treatment did not alter immunostaining. Panel (**d**) are representative images of eNOS staining. Data are presented as mean ± SEM. Two-way (testosterone treatment and vitamin D status) ANOVA; d: *p* < 0.05 D+ vs. D-. N = 4–6 in each group. Panel (**e**) Immunohistochemical staining of the aortic wall with COX-2 antibody. Percentage is calculated by comparing the positively stained area to total tissue area. Testosterone treatment significantly increased COX-2 staining in our experiment. D+T- group had significantly larger area stained compared to control. Panel (**f**) shows COX-2 staining in the different groups. Data are presented as mean ± SEM. Two-way (testosterone treatment and vitamin D status) ANOVA; Tukey’s post hoc test, $: *p* < 0.05 T-D+ vs. T+D+ group, t: *p* < 0.05 D+ vs. D-. N = 4–5 in each group.

In general, testosterone treatment increased while VDD decreased estrogen receptor (ER) specific labeling in the aortic intimal layer. Post hoc test revealed significantly decreased ER staining in the T-D- group compared to all other groups (Figure 3 Panel (a)). VDD resulted in significantly reduced eNOS specific staining intensity in the examined vessels according to two-way ANOVA. However, testosterone treatment failed to influence it. No intergroup difference was confirmed by post hoc test (Figure 3 Panel (c)). COX-2 specific labeling showed significant increase after testosterone treatment. Post hoc testing revealed a significant elevation in the T+D+ group compared to control animals. (Figure 3 Panel (e)).

## 3. Discussion

Many experimental rat models are found in the literature, which aims to achieve hyperandrogenic state using various chronic treatments [19,20,21,22,23,24,25,26,27]. Our aim was to examine the effect of VDD in a moderate hyperandrogenic milieu-hyperandrogenism was induced by a relatively short, 8-week-long transdermal testosterone treatment.

In our experiment, the induced hyperandrogenic state together with vitamin D deficiency (T+D-) was appropriate to examine a PCOS-like condition [15,16].

Endothelial dysfunction is a well-characterized phenomenon in women with PCOS that contributes to the higher cardiovascular risk of these patients [3,28]. In our other experiment, norepinephrine-induced vasoconstriction was not influenced by either androgenic or vitamin D status. However, endothelial dysfunction was detected in VDD groups in the form of reduced acetylcholine-mediated vasorelaxation. Furthermore, local insulin resistance in the aortic rings caused a significantly reduced insulin-dependent vasorelaxation at higher insulin doses in the VDD groups [15]. In the present study, we examined the estradiol-induced vasorelaxation in thoracic aorta segments of female rats. Estradiol can act not only through nuclear ERα and ERβ receptors, but it can also act through a non-genomic pathway via G protein-coupled estrogen rseceptor (GPER), all of which can contribute to its vasorelaxant effect [29]. Bucci et al. described that in male rats the main pathway for the estradiol-induced vasorelaxation is linked to both eNOS phosphorylation through Akt/PkB pathway and higher PGI_2_ levels [30]. PGI_2_ synthesis involves both COX-1 and COX-2 enzymes, the latter being directly affected by estrogen [7].

In our experiments, decreased estradiol-dependent vasorelaxation was measured in the VDD groups (T-D-, T+D-). VDD also resulted in reduced ER expression of endothelial cells that was significantly different in the T-D- group compared to the T-D+ rats. Despite the assumption based on our previous study, we failed to confirm the similar effect of chronic testosterone treatment on vascular function [14]. We believe that a shorter, only 8-week-long hyperandrogenic state was sufficient to induce PCOS phenotype as diagnosis according to Rotterdam criteria relies on early signs of the disease. However, it failed to initiate endothelial dysfunction which is primarily considered to be a long-term complication. On the other hand, VDD seems to act more rapidly in terms of deteriorating vessel function. Normal serum vitamin D level is essential for the corresponding vascular function. It is well known that VDD increases the risk of cardiovascular diseases (CVD) and hypertension [31].

To examine the main mechanism of estradiol-dependent vasorelaxation, L-NAME was used to block eNOS. After L-NAME incubation, decreased vasorelaxation was measured in every group, confirming the importance of NO production in this mechanism. VDD significantly reduced eNOS staining according to our results. As it was described earlier, vitamin D could activate eNOS activity through the PI3K/protein kinase B (Akt) pathway [12]. Previous in vitro cell culture and animal models demonstrated that the lack of vitamin D effect leads to the eNOS mRNA downregulation. The effect of vitamin D on arginase II enzyme may also contribute to reduce NO production in these circumstances. Vitamin D inhibits arginase II that competes with eNOS for their common substrate, L-arginine [12,32].

Little is known about the changes in prostanoids production in other organs. In arteries, prostanoids play an important role in physiological vascular responses. TXA_2_, PD_2_, PE_2_ and PF_2α_ can induce vasoconstriction. Prostacyclin (PGI_2_) through its own receptor mainly evokes vasorelaxation. In female rats, COX2 inhibitor increased the acetylcholine–induced vasorelaxation. Moreover, higher COX-2 levels were found in the ovaries of PCOS rats [33]. There are gender differences in the vascular relaxation after incubation with selective or non-selective COX inhibitors. The COX-2 inhibitor has a pronounced effect in female rats, while in male rats the inhibitor effect of the indomethacin (which acts as a general inhibitor of COX isoforms), led to the same results. Data from earlier studies suggest that this phenomenon can be explained by estrogen mediating the enhancement of COX-2 expression [13,34,35].

Although we were unable to show a significant increase in COX-2 staining in VDD due to pronounced parallel effect of testosterone treatment, the increased significance of the COX-2 pathway was demonstrated by the decreased estrogen-induced vasodilation during COX-2 inhibition in the VDD groups. The level of end products of COX-2 is also influenced by phospholipase A2 (PLA2) expression and activity which itself is also regulated by vitamin D [36]. Hyperandrogenism in female rodents was demonstrated to induce COX-2 expression in reproductive tissues [37,38]. We found a similar effect of testosterone-treatment in the aortic endothelial cells in our experiments. However, blocking COX-2 did not deteriorate estrogen-induced vasodilatation. COX-2 pathway has several vasoconstrictor end products as well, including TXA_2_. Expression of TXA_2_ synthase is upregulated by chronic testosterone treatment in female rat cerebral arteries [39]. A change in the equilibrium of vasoconstrictor and vasodilator eicosanoids could explain our results.

## 4. Materials and Methods

### 4.1. Chemicals

Functional measurement on isolated rat aortas was performed in freshly prepared normal Krebs–Ringer (nKR) solution (in mmol/L): NaCl 119, KCl 4.7, NaH_2_PO_4_ 1.2, MgSO_4_ 1.17, NaHCO_3_ 24, CaCl_2_ 2.5, glucose 5.5 and EDTA 0.034. These solutions (Sigma-Aldrich (St. Louis, MO, USA; Budapest, Hungary) were kept at 37 °C, and bubbled with a gas mixture (containing O_2_ 20%, CO_2_ 5% and N_2_ 75%) to stabilize pH (Linde Ltd., Répcelak, Hungary).

17-β -estradiol was purchased from TOCRIS Bio-Techne (Bristol, UK), while testosterone, eNOS inhibitor (L-NAME, 10^−4^) and COX-2 inhibitor (NS398, 10^−5^) was acquired from Sigma-Aldrich (St Louis, MO, USA).

### 4.2. Animals

Forty-six adolescent (21–28 day-old), 100–140 g-weighing female Wistar rats were delivered to the Animal Facility of Semmelweis University in agreement with Charles River (Charles River Ltd., AnimaLab, Vác, Hungary). The animals were randomly assigned to four experimental groups; hyperandrogenic vitamin D-deficient group (T+D-, N = 11); hyperandrogenic vitamin D-supplemented group (T+D+, N = 12); vitamin D-deficient group (T-D-, N = 11) and vitamin D-supplemented group (T-D+, N = 12). The institutional Animal Care Commission has confirmed the research protocol (IRB: 8/2014 PEI/001/1548-3/2014).

### 4.3. Chronic Treatment of the Rats

Hyperandrogenism was induced by an 8-week-long transdermal testosterone treatment applying 0.0333 mg/g of Androgel (50 mg/5 mL gel by Lab. Besins International S.A., Paris, France) 5 times a week on a previously shaved 3 × 3 cm area on the back of the animals [40].

**Figure 4:**
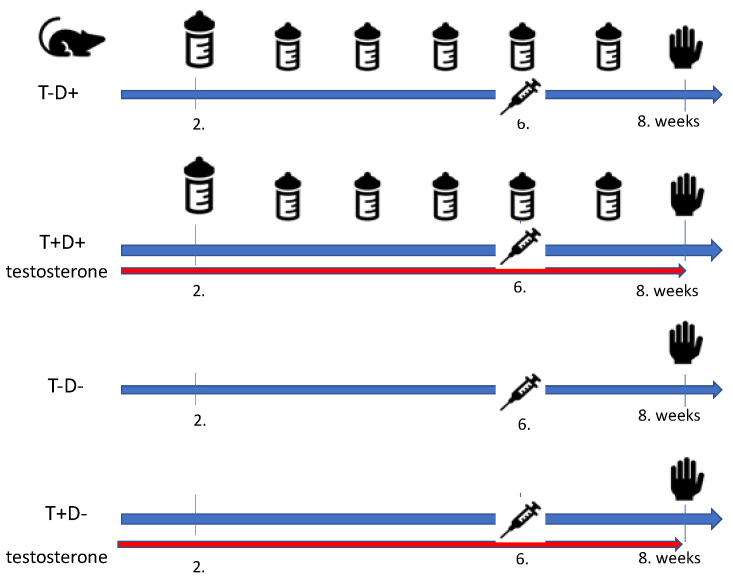
Timeline of chronic treatment. Adolescent Wistar rats were grouped as the following: half of the animals (T+D+, T+D-) received transdermal testosterone treatment (0.0333 mg/body weight grams 5 times weekly) for 8 weeks. Half of the testosterone-treated and half of the non-treated animals fed low vitamin D chow (T-D-, T+D-) while the rest received adequate vitamin D supplementation (weekly 1,4 NE/body weight grams per os on the 3rd, 4th, 5th, 6th and 7th week after a 500NE saturation on the 2nd week) (T-D+, T+D+). The animals were sacrificed on the 8th treatment week and ex vivo experiments were performed.

VDD was generated by reduced vitamin D intake the animals being fed with Vitamin D Free Lab Rat/Mouse Chow (Ssniff Spezialdiaten GmbH, Soest, Germany) containing less than 5 IU/kg vitamin D_3_. Supplemented animals had access to a regular chow containing 1000 IU/kg of vitamin D_3_. Vitamin D-supplemented animals received additional oral vitamin D supplementation as the following: 500 IU cholecalciferol on the second week and a weekly dose of 140 IU/100 g on the fifth, sixth and seventh weeks (Vigantol (cholecalciferol) 20,000 IU/mL, Merck/Merck Serono, Darmstadt, Germany) using a gavage cannula. According to the human vitamin D supplementation guidelines, the target serum 25-hydroxy-cholecalciferol levels were optimized to 30–50 ng/mL [16,18]. The animals had access to the appropriate amount of rat chow and tap water *ad libitum*. Rats were housed at constant room temperature (22 ± 1 °C) in 12 h/12 h light-dark cycle. Figure 4 shows an outline of the chronic treatment of different groups.

After the 8-week-long treatment rats were anesthetized with Nembutal (45 mg/kg intraperitoneal). Before removing the thoracic aorta, the cardiovascular system was transfused with heparinized nKR solution for 2 min. The carefully prepared aortic segment was cut into 9 (3 mm long) equal pieces. 8 of these were placed on a conventional wire myograph setup (610-M MultiMyograph System, Danish Myo Technology, Hinnerup, Denmark/AnimaLab, Hungary). The 9th aorta ring was fixed in formalin and embedded in paraffin.

### 4.4. Myography

A conventional wire myograph system was used to measure the isometric tension of isolated thoracic aortic rings. The organ chambers were filled with 8 mL nKR and kept at 37 °C. 15 mN pre-tension was reached progressively. After the development of stable pre-tension, 124 mmol/L K^+^ was applied (3 min) to test the contractility of the vessels and to serve as the reference value for contraction force. The aortic rings then were equilibrated in nKR and precontraction was generated by norepinephrine (5 × 10^−8^ mol/L). Integrity of the endothelium was checked using acetylcholine 10^−7^ mol/L. Estradiol relaxation was examined in 3 different doses (10^−7^–10^−5^ mol/L) after 5 × 10^−8^ norepinephrine precontraction. To identify possible endothelium-dependent relaxing pathways the following inhibitors were used before the 2nd and 3rd precontraction (L-NAME 10^−4^ M and NS398, 10^−5^ M).

### 4.5. Immunohistochemistry

Paraffin-embedded tissue sections were stained against estrogen receptor alpha (ERα), endothelial nitric oxide synthase (eNOS), cyclooxygenase 2 (COX-2). After deparaffinization antigen retrieval was performed by heating the slides in citrate buffer (pH = 6). Endogenous peroxidase activity was blocked by 10% H_2_O_2_ in dH_2_O. 2.5% normal horse serum (Vector Biolabs, Burlingame, CA, USA) was used to avoid aspecific labeling. Primary antibodies (COX-2: 1:500, eNOS: 1:50 (Abcam, Cambridge, UK)) were applied overnight at 4 °C. HRP-linked anti-mouse or anti-rabbit polyclonal horse antibody (Vector Biolabs, Burlingame, CA, USA) was used for secondary labeling. Brown-colored diamino-benzidine (DAB) was used for the visualization of specific labeling (Vector Biolabs, Burlingame, CA, USA). Blue-colored hematoxylin served as counterstaining (Vector Biolabs, Birmingham, CA, USA). ER immunohistochemistry was performed using BenchMark ULTRA Automated IHC/ISH slide staining system (primary antibody 1:100) (Ventana Medical Systems, Inc., Tucson, AZ, USA). Visualization of specific labeling with DAB as colored substrate and hematoxylin counterstaining was achieved by UltraView Universal DAB Detection Kit (Ventana Medical Systems, Inc., Oro Valley, AZ, USA). Light microscopy images were taken with Zeiss Axio Imager system (Zeiss, Oberkochen, Germany). The positively stained area as a percentage of total tissue area or non-calibrated optical density of specific staining was measured in the intimal and medial layers of the vessel walls using the ImageJ software (National Institutes of Health (NIH), Bethesda, MA, USA).

### 4.6. Statistics

Effect of testosterone treatment and vitamin D status were evaluated by two-way ANOVA using Tukey’s post-hoc test by Prism 8 (GraphPad, GraphPad Software, USA). Vascular function curves were analyzed by repeated measures two-way ANOVA using Bonferroni’s post-hoc test. *p* < 0.05 was uniformly accepted as the threshold for statistical significance.

## 5. Conclusions

In our rodent model of PCOS, we examined the possible early vascular changes of the disease. We examined the possible interplay of two characteristic findings in PCOS: hyperandrogenism and vitamin D deficiency.

In this relatively short-term treatment period, we were not able to detect endothelial dysfunction in the hyperandrogenic group.

However, VDD, which is a common comorbidity in PCOS, induced marked vascular dysfunction.

This endothelial dysfunction was accompanied by decreased ER and eNOS immunostaining. We observed other initial alterations causing endothelial dysfunction and playing key roles in target organ damages observed in PCOS, e.g., increased COX activation and elevated ROS levels.

Consequently, treatment of VDD may delay the onset of arising endothelial dysfunction and consequent increase in cardiovascular risk. Previous studies also confirmed that vitamin D treatment is beneficial in the normalization of hormonal disturbances in PCOS women [41]. Considering our results, adequate vitamin D supplementation can be elementary beyond the menstrual cycle regulation to alleviate the pathological vascular adaptation in early PCOS.

## Data Availability

Study data are provided in Supplementary Materials.

## Supplementary Materials

The following are available online at https://www.mdpi.com/article/10.3390/ijms22179404/s1.
