# Supplementary material for: Influence of Vitamin D on the Vasoactive Effect of Estradiol in a Rat Model of Polycystic Ovary Syndrome
Source: Int J Mol Sci. 2021 Aug 30;22(17):9404. doi: 10.3390/ijms22179404 (PMC8431242; doi:10.3390/ijms22179404)
Supplement: Supplementary file 1 [file ijms-22-09404-s001.zip › ijms-1345992-supplementary.pdf]

Figure S1. Estradiol dependent vasorelaxation

T-D+				T+D+		
	0.0000001	0.000001	0.00001	0.0000001	0.000001	0.00001
	0.750	5.990	2.890	43.630	48.380	49.210
	54.01	27.360	11.400	27.940	29.160	25.010
	11.610	30.670	25.610	36.530	44.380	47.230
	8.480	11.110	7.440	16.590	17.630	15.390
	13.380	16.500	19.990	32.720	32.270	29.630
	11.550	18.620	17.450	16.710	18.330	18.330
	48.650	46.640	42.980	31.580	36.720	40.250
	27.320	35.970	38.490	41.860	51.470	55.810
	11.370	14.880	13.490	21.920	28.560	34.690
	6.760	14.110	24.000	29.600	46.210	52.380
	7.570	10.950	14.330	37.190	47.670	48.130
	20.590	28.680	34.190	14.950	23.850	24.020
	4.440	0.890	-5.420	23.160	30.010	31.120
	14.230	15.360	14.230	26.370	36.550	45.620
	26.970	44.980	72.340	27.020	34.350	38.180
	31.550	48.730	57.930	41.340	40.180	42.380
	40.480	57.140	66.330	35.060	46.740	49.250
	19.200	34.410	52.560	25.460	36.130	40.190
	28.260	36.740	38.040	32.440	46.370	49.880
	22.140	32.140	40.320	30.660	44.140	51.730
	30.830	41.600	50.630	13.820	23.530	35.150
	16.580	21.920	23.250	37.790	46.140	55.560
	10.810	16.870	20.820	21.120	24.300	23.420
	16.970	24.860	28.880	8.9608.960	11.880	15.400
	13.620	19.900	26.930	27.240	35.750	45.530
	8.270	11.500	25.370	1.8301.830	5.930	26.770
	12.830	20.130	32.080	0.7500.750	-0.480	-0.480
	21.030	38.530	37.650	9.3709.370	19.850	29.970

22.760	32.140	40.240	17.420	23.970	36.050

Figure S1. Estradiol dependent vasorelaxation

T-D+			T+D+		
0.0000001	0.000001	0.00001	0.0000001	0.000001	0.00001
17.260	19.350	-1.490	6.9606.960	21.380	42.630
10.030	16.740	26.310	9.4409.440	19.980	39.670
17.120	23.890	25.990			
6.090	9.100	6.810			

Figure S1. Estradiol dependent vasorelaxation

T-D-

0.0000001	0.000001	0.00001
2.670	5.450	5.870
3.370	5.050	6.150
4.290	5.490	3.570
2.500	1.190	-3.140
-0.080	-3.710	-0.770
1.240	1.460	-16.440
2.610	-1.550	-4.230
6.350	8.870	12.060
3.150	2.310	2.240
2.390	-4.310	-13.410
4.260	0.410	-1.540
9.780	12.660	11.810
14.830	17.880	22.760
26.100	34.130	42.500
19.020	37.490	66.760
-1.030	9.090	21.630
22.960	36.340	41.170
20.290	14.910	25.350
14.780	25.190	46.560
9.700	11.400	6.870
35.090	48.850	54.430
35.720	48.230	52.050
17.420	21.870	24.390
5.540	10.360	13.360
11.570	15.760	22.540
15.850	28.560	35.880

21.210	28.260	47.240
20.530	29.460	33.570
14.220	19.990	26.170

Figure S1. Estradiol dependent vasorelaxation

T-D-

0.0000001	0.000001	0.00001
25.120	35.910	39.980
19.210	33.700	30.660
9.570	7.370	4.100
4.670	-15.540	-29.440
15.790	22.510	22.340
7.530	9.260	12.100
6.340	7.430	8.810
5.770	10.860	12.940
15.830	25.470	37.970
2.900	1.320	-2.770
6.450	-0.410	-14.650
9.690	10.080	9.330
27.760	42.510	62.040

Figure S1. Estradiol dependent vasorelaxation

T+D-

0.0000001	0.000001	0.00001
12.580	14.650	13.150
11.170	14.630	12.520
27.060	36.970	45.240
17.130	27.160	34.600
10.250	15.150	14.750
16.610	25.190	28.650
24.380	26.990	29.850
17.370	20.870	23.590
20.320	22.940	22.460
21.400	22.560	20.320
6.900	9.560	9.130
14.940	17.640	18.340
11.990	11.240	22.540
18.750	21.710	19.990
16.270	21.760	54.710
5.040	2.020	7.350
5.020	1.870	1.280
0.860	-4.700	-9.470
8.450	15.850	10.710
6.640	3.330	2.760
10.490	11.000	4.370
5.570	3.590	-1.300
13.970	23.560	21.650
27.500	38.660	39.640

3.970	4.710	5.410
39.600	37.650	49.020
12.220	17.640	17.670
23.380	24.890	22.050
16.160	26.470	26.470

Figure S1. Estradiol dependent vasorelaxation

T+D-

0.0000001	0.000001	0.00001
13.220	21.640	21.640
7.600	10.200	5.420
7.340	8.810	8.710
16.980	27.100	26.770
14.240	21.230	18.070
10.080	11.130	11.130
15.450	15.450	10.550
25.290	21.780	17.420
16.970	26.210	31.860
30.490	30.880	25.040

Figure S2. Estradiol-induced vasorelaxation in the presence of eNOS (L-NAME) or COX-2 (NS398) inhibitors.

Panel (a): T-D+ group

without inhibition				L-NAME		
	0.0000001	0.000001	0.00001	0.0000001	0.000001	0.00001
	36.52	34.58	27.17	0.120.120.12	-8.11	-13.33
	8.99	-3.87	-19.02	1.731.731.73	-6.22	-18.37
	15.65	18.86	10.5	7.497.497.49	10.99	11.97
	17.35	18.3	19.24	3.623.623.62	-0.79	-0.13
	24.48	28.62	28.62	1.681.681.68	-7.3	-25.48
	15.63	25.22	33.73	2.822.822.82	0.17	8.97
	7.55	-0.37	-14.92	11.6811.68	12.4	15.36
	25.73	34.11	39.38	0.4600	-4.79	-9.58
	7.75	6.14	16.12			
	16.54	23.31	32.71			
	24.78	24.34	50.88			
	8.2	4.04	35.01			
	2.15	0.21	0.35			

Figure S2. Estradiol-induced vasorelaxation in the presence of eNOS (L-NAME) or COX-2 (NS398) inhibitors.

Panel (b): T+D+ group

without inhibition				L-NAME		
	0.0000001	0.000001	0.00001	0.0000001	0.000001	0.00001
	51.44	44.13	22.03	15.25	9.24	-1.59
	32.02	35.08	35.92	11.13	9.29	6.33
	24.8	82.38	-1.43	10.59	13.79	15.77

74.29	74.9	72.24	3.39	0	-5.74
87.65	95.06	-6.17	6.22	-13.87	-29.71
-1.13	-12.39	-30.52	1.94	-11.56	-2.66
30.3	33.61	35.26	5.17	6.89	34.85
23.13	40.36	36.96			
18.69	26.69	24.23			
26.92	36.86	41.67			

Figure S2. Estradiol-induced vasorelaxation in the presence of eNOS (L-NAME) or COX-2 (NS398) inhibitors.

Panel (b): T+D+ group

without inhibition

0.0000001	0.000001	0.00001
9.98	15.73	24.5
43.46	48.36	52.04
6.76	4.2	8.05
6.73	7.6	20.71

Figure S2. Estradiol-induced vasorelaxation in the presence of eNOS (L-NAME) or COX-2 (NS398) inhibitors.

Panel (c): T-D- group

without inhibition			L-NAME		
0.0000001	0.000001	0.00001	0.0000001	0.000001	0.00001
5.66	6.73	5.99	2.13	-1.87	-10.3
3.58	-3.77	-12.24	-0.19	-3.11	-8.02

6.72	2.21	-2.55	0.9	-0.77	-1.51
3.42	-2.78	-6.61	5.22	-2.01	-17.47
8.67	7.43	7.56	9.83	2.58	-3.05
18.8	18.8	8.94	0.29	-13.68	-18.48
12.5	14.42	34.03	3.63	-3.81	-9.96
33.72	38.4	36.84	14.59	11.77	0.67
16.3	24.22	38.11	5.5	-12.5	-38.08
10.46	11.24	11.41	3.51	4.52	5.81

Figure S2. Estradiol-induced vasorelaxation in the presence of eNOS (L-NAME) or COX-2 (NS398) inhibitors.

Panel (c): T-D- group

without inhibition

0.0000001	0.000001	0.00001
6.58	6.06	5.11
20.14	16.47	15.06
21.18	33.33	48.63
28.77	28.91	50.28
8.1	26.3	47.87
7.85	-2.7	-14.71
23.34	36.15	40.76
3.95	-6.83	-12.19
38.26	45.4	59.54

Figure S2. Estradiol-induced vasorelaxation in the presence of eNOS (L-NAME) or COX-2 (NS398) inhibitors.

Panel (d): T+D- group

without inhibition

0.0000001	0.000001	0.00001
16.17	19.72	19.54
19.42	27.44	27.76
7.28	7.65	4.63
22.15	25.49	48.82
27.79	21.96	21.96
27.98	23.46	22.7
4.14	-3.69	-11.87
13.21	19.8	19.32
58.3	61.8	69.5
36.8	35.88	35.31
2.39	-4.45	-10.84
39.37	43.71	40.83
20.57	16.43	-0.39
16.08	37.25	42.94
13.2	18.35	20.36
15.03	36.59	42.39
18.23	13.7	3.04
18.6	24.01	29.94

L-NAME

0.0000001	0.000001	0.00001
8.25	9.35	17.75
7.11	2.09	-0.51
21.65	34.96	37.84
0.25	-24.77	-41.65
0.04	-1.99	-6.3
34.91	41.85	44.42
7.92	14.03	7.95
3.31	-5.84	-29.77
2.66	-13.25	-30.7
7.9	3.68	-5.52
-3.34	-19.77	-43.47

23.63	32.57	24.82

Figure S3. Immunohistochemical changes in thoracic aorta wall.

Panel (a) ER

Vitamin D supplementation		Vitamin D deficiency	
Control	Testosterone	Control	Testosterone
0.135409	0.14766	0.130206	0.156888
0.162726	0.15734	0.109438	0.151715
0.150918	0.130959	0.105076	0.17532
0.150428	0.152817	0.098695	0.116862
0.141466	0.171425	0.090132	0.153379
	0.206645	0.061478	0.15278
			0.042282

Figure S3. Immunohistochemical changes in thoracic aorta wall.

Panel (c) eNOS

Vitamin D supplementation		Vitamin D deficiency	
Control	Testosterone	Control	Testosterone
0.297826	0.292462	0.308594	0.034581
0.325377	0.063908	0.080823	0.048423
0.218497	0.160219	0.088821	0.315507
0.3695	0.27844	0.132585	0.182961
0.243554			
0.197228			

Figure S3. Immunohistochemical changes in thoracic aorta wall.

Panel (e) COX-2

Vitamin D supplementation		Vitamin D deficiency	
Control	Testosterone	Control	Testosterone
15.01745	20.33375	21.98428	18.87836
11.07665	46.53739	12.51002	28.76419
5.382877	29.9327	29.49834	6.53123
5.606379	76.79092	40.534	37.06894
11.97878		10.92393	

Figure S2. Estradiol-induced vasorelaxation in the presence of eNOS (L-NAME) or COX-2 (NS398) inhibitors.

Panel (a): T-D+ group

COX-2 inhibitor

0.0000001	0.000001	0.00001
17.38	25.31	28.72
5.63	10.04	-7.6
11.52	7.37	10.83
19.5	24	18
49.4	24.1	-6.02
20.83	29.52	53.44
8.26	9.09	-3.58
14.35	37.31	29.96
5.10	11.86	2.77

Figure S2. Estradiol-induced vasorelaxation in the presence of eNOS (L-NAME) or COX-2 (NS398) inhibitors.

Panel (b): T+D+ group

COX-2 inhibitor

0.0000001	0.000001	0.00001
16.58	19.17	19.89
23.43	43.51	55.37
13.52	18.51	12.99

6.02	17.78	50.59
2.47	2.47	2.04
39.83	44.82	60.11

Figure S2. Estradiol-induced vasorelaxation in the presence of eNOS (L-NAME) or COX-2 (NS398) inhibitors.

Panel (c): T-D- group

COX-2 inhibitor		
0.0000001	0.000001	0.00001
6.54	6.6	15.32
-1	-4.88	-2.53

17.81	12.1	-1.3
-2.25	7.31	-8.48
3.88	1.35	-9.03
1.32	-9.04	-16.93
13.74	10.26	9.74
5.42	-2.68	-15.19
10.78	6.54	-5.61
3.1	-3.91	-5.39

Figure S2. Estradiol-induced vasorelaxation in the presence of eNOS (L-NAME) or COX-2 (NS398) inhibitors.

Panel (d): T+D- group

COX-2 inhibitor		
0.0000001	0.000001	0.00001
18.15	12.85	9.96
13.83	-27.88	-60.71
8.92	1.3	30.93
49.49	52.51	69.33
12.46	8.79	-1.58
9.69	3.07	-7.66
1.23	-11.58	-26.2
8.1	8.71	-20.81
17.39	-10.13	-36.9
